# Supplementary material for: A Minimal PBPK/PD Model with Expansion-Enhanced Target-Mediated Drug Disposition to Support a First-in-Human Clinical Study Design for a FLT3L-Fc Molecule
Source: Pharmaceutics. 2024 May 15;16(5):660. doi: 10.3390/pharmaceutics16050660 (PMC11125320; doi:10.3390/pharmaceutics16050660)

Figure S2: Projected time profiles of clinical PK, cDC1, and total DC cells in healthy volunteers treated with a single dose of FLT3L-Fc

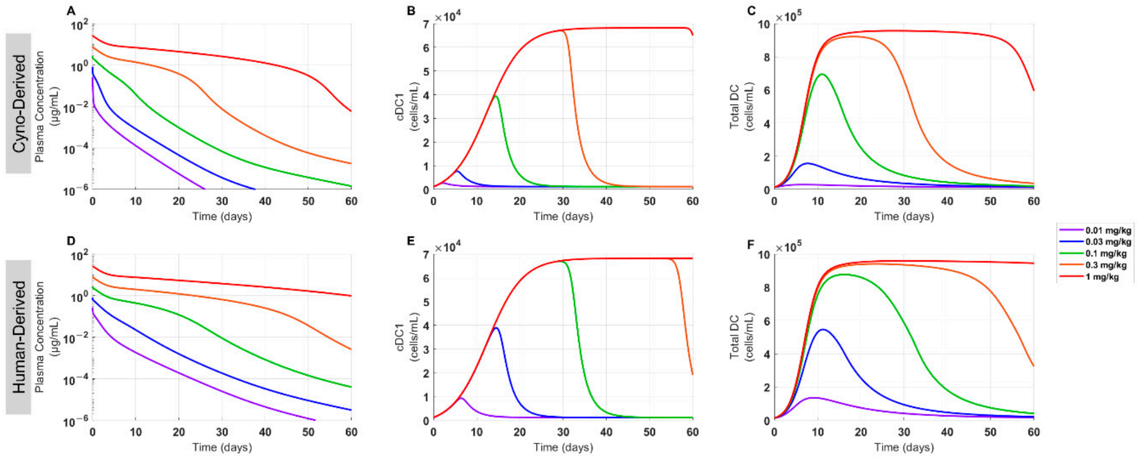

Supplement: Supplementary file 1 [file pharmaceutics-16-00660-s001.zip › FLt3L_Fc_manuscript_pharmaceutics_supp-002-figure-S2 - Done.pdf]
