# Supplementary material for: A Minimal PBPK/PD Model with Expansion-Enhanced Target-Mediated Drug Disposition to Support a First-in-Human Clinical Study Design for a FLT3L-Fc Molecule
Source: Pharmaceutics. 2024 May 15;16(5):660. doi: 10.3390/pharmaceutics16050660 (PMC11125320; doi:10.3390/pharmaceutics16050660)
Supplement: Supplementary file 1 [file pharmaceutics-16-00660-s001.zip › FLt3L_Fc_manuscript_pharmaceutics_supp-004_tableS1 - Done.pdf]

Table S1: Overview of clinical and preclinical studies used for model calibration/validation

Study Title	Dose/ Regimens	Measurements	Data Source
Preclinical Study 1: Single-Dose and Repeat-Dose of FLT3L-Fc in Cynomolgus Monkeys	Single IV dose of FLT3L-Fc at 0.1 mg/kg; repeat IV doses at 1 mg/kg or 10 mg/kg on days 0 and 21 (n=3)	PK; PK sampling schedule: 0.02, 0.08, 0.25, 1, 3, 7, 9, 14, 21, 21.02, 21.08, 21.25, 22, 24, 28, 30, and 35 days	Genentech Study
Preclinical Study 2: Repeat-Dose of FLT3L-Fc in Cynomolgus Monkeys	Repeat IV dose of FLT3L-Fc at 1 or 3 mg/kg on days 0 and 21 (n=6)	PK. PK sampling schedule: 0.02, 0.08, 0.25, 1, 3, 7, 9, 14, 21, 21.02, 21.08, 21.25, 22, 24, 28, 30, and 35 days	Genentech Study
Clinical Study 1: Phase I Clinical Trial of CDX-301 in Healthy Volunteers*	Repeat daily SC dose of CDX-301 dose at 1, 3, 10, 25 or 75 µg/kg for 5 days, 25 µg/kg for 7 days, or 25 µg/kg for 10 days	PK; multiple PD biomarkers such in peripheral blood; see Anandasabapathy N. et al for the sampling schedule	Ref. (1)
Clinical Study 2, Part 1: recombinant FLT3L in Healthy Volunteers*	Repeat daily SC dose of recombinant FLT3L at 100 µg/kg for 14 days	PK; multiple PD biomarkers in peripheral blood; see Maraskovsky E. et al for the sampling schedule	Ref. (2)
Clinical Study 2, Part 2: recombinant FLT3L in Healthy Volunteers*	Repeat daily SC dose of recombinant FLT3L at 10, 25, 50, 75, or 100 µg/kg for 14 days	PK; multiple PD biomarkers in peripheral blood; see Maraskovsky E. et al for the sampling schedule	Ref. (2)
Clinical Study 3: Phase I Clinical Trial of GS-3583 in Healthy Volunteers*	Single IV dose of GS-3583 at 225 or 675 µg	PK; see Rajakumaraswamy N. et al for the sampling schedule	Ref. (3)

*Digitized data from source publication

References

1. Anandasabapathy, N.; Breton, G.; Hurley, A.; Caskey, M.; Trumpfheller, C.; Sarma, P.; Pring, J.; Pack, M.; Buckley, N.; Matei, I.; et al. Efficacy and safety of CDX-301, recombinant human Flt3L, at expanding dendritic cells and hematopoietic stem cells in healthy human volunteers. *Bone Marrow Transpl.* **2015**, *50*, 924–930. https://doi.org/10.1038/bmt.2015.74.
2. Maraskovsky, E.; Daro, E.; Roux, E.; Teepe, M.; Maliszewski, C.R.; Hoek, J.; Caron, D.; Lebsack, M.E.; McKenna, H.J. In vivo generation of human dendritic cell subsets by Flt3 ligand. *Blood* **2000**, *96*, 878–884.
3. Rajakumaraswamy, N.; Dauki, A.; Kuhne, M.R.; Trowe, T.; He, J.; Clarke, C.; Carr, B.; Worth, A.; Vashishtha, A.; Schwabe, C.; et al. GS-3583, a novel FLT3 agonist Fc fusion protein, to expand conventional dendritic cells in healthy volunteers. *J. Clin. Oncol.* **2021**, *39*, 2559. https://doi.org/10.1200/JCO.2021.39.15_suppl.2559.
