# Supplementary material for: A Minimal PBPK/PD Model with Expansion-Enhanced Target-Mediated Drug Disposition to Support a First-in-Human Clinical Study Design for a FLT3L-Fc Molecule
Source: Pharmaceutics. 2024 May 15;16(5):660. doi: 10.3390/pharmaceutics16050660 (PMC11125320; doi:10.3390/pharmaceutics16050660)
Supplement: Supplementary file 1 [file pharmaceutics-16-00660-s001.zip › FLt3L_Fc_manuscript_pharmaceutics_supp-005_tableS2 - Done.pdf]

Table S2: Model parameters

Parameter (unit)	Definition	Cyno Value	Human Value	Source
MW_{FC} (Da)	Molecular weight of FLT3L-Fc	83000	83000	In-house measurement
MW_{CDX} (Da)	Molecular weight of CDX-301	35000	35000	Ref. (1)
BW (kg)	Body weight	2.6	70	Nominal body weight value for species
Vlymph (L)	Volume of lymph	0.086	5.2	Cyno fit from data, human from (2)
Vplasma (L)	Volume of plasma	0.09	2.6	Cyno fit from data, human from (2)
CLp (L/h)	Nonspecific clearance of FLT3L-Fc	4.2E-04	6.94E-03	Cyno fit from data, human allometrically scaled
CLp (mL/d/kg)	Nonspecific clearance of FLT3L-Fc	3.87	2.4	Cyno fit from data, human allometrically scaled
σ_{leaky}	Leaky reflection coefficient	0.656	0.656	Fit from data
σ_{tight}	Tight reflection coefficient	0.985	0.985	Fit from data
σ_{lymph}	Lymph reflection coefficient	0.2	0.2	Ref. (2)
L (L/h)	Lymph flow rate	0.012	0.121	Cynomolgus monkey fit from data, human from (2)
Kp	Partition coefficient	0.8	0.8	Ref. (2)
ISF (mL/kg)	Interstitial fluid	0.579	15.6	Cynomolgus monkey fit from data, human from (3)
f_{leaky}	Fraction of leaky flow from blood to leaky	0.65	0.65	Ref. (2)
f_{tight}	Fraction of lymph flow from blood to tight	0.35	0.35	Ref. (2)
$C_{R,0}$ (nM)	Initial concentration of FLT3 receptors in circulation	11.3	4	Fit from data
vm_{prolif} (h^{-1})	Maximum rate of FLT3 expansion induced by double-bound FLT3 receptor-drug complex	1.90E-03	3.30E-3	Fit from data
km_{prolif}	Amount of double-bound FLT3 receptor-drug complex to achieve 50% vm_{prolif}	0.547	0.02	Fit from data
α	Hill coefficient of FLT3 expansion induced by double-bound FLT3 receptor-drug complex	3.93	1	Fit from data
kdeg (h^{-1})	Turnover rate of FLT3 receptors in circulation	0.016	0.016	Fit from data
kon1 (1/nM/h)	On-rate for monovalent binding of drug to FLT3 receptors	0.1	0.1	Assumption
KD1 (nM)	monovalent binding affinity of drug to FLT3 receptors	9	9	Ref. (4)
kon2 (1/nM/h)	On-rate for binding of single-bound drug-FLT3 complex to the second FLT3 receptor	3.6	3.6	in-house Biacore measurement
KD2 (nM)	Binding affinity of single-bound drug-FLT3 complex to the second FLT3 receptor	0.2	0.2	in-house Biacore measurement
$kabs_{CDX}$ (h^{-1})	Absorption rate of CDX-301 from SC to plasma	NA	0.04	Fit from CDX-301 data (5)

Vd_{CDX} (mL/kg)	Volume of distribution of CDX-301	NA	213	Fit from CDX-301 data (5)
vm_{CDX} (mL/h/kg)	Maximum nonlinear elimination rate of CDX-301	NA	3.72	Fit from CDX-301 data (5)
km_{CDX} (µg/mL)	Concentration of CDX-301 needed to achieve 50% of Vm_{CDX}	NA	0.394	Fit from CDX-301 data (5)
$init_{DC1}$ (cells/mL)	Mean initial cDC1 count	NA	1180	Human estimated from (5)
$kdeg_{DC1}$ (h^{-1})	Turnover rate of the cDC1 population	NA	1.60E-02	Fit from CDX-301 data (5, 6)
$vm1_{DC1}$ (h^{-1})	Maximum rate of cDC1 expansion induced by free drug	NA	1.61	Fit from CDX-301 data (5, 6)
$km1_{DC1}$ (nM)	Concentration of free drug to increase cDC1 counts to 50% $Vm1_{DC1}$	NA	0.072	Fit from CDX-301 data (5, 6)
del_{DC1} (h^{-1})	Transit rate between cDC1 compartments	NA	0.114	Fit from CDX-301 data (5, 6)
$n1_{DC1}$	Hill coefficient of free drug effect on cDC1 expansion	NA	5.85	Fit from CDX-301 data (5, 6)
$init_{DC2}$ (cells/mL)	Mean initial cDC2 count	NA	12700	Human estimated from (6)
$kdeg_{DC2}$ (h^{-1})	Turnover rate of the cDC2 population	NA	1.70E-03	Fit from CDX-301 data (5, 6)
$vm1_{DC2}$ (h^{-1})	Maximum rate of cDC2 expansion induced by free drug	NA	16.1	Fit from CDX-301 data (5, 6)
$km1_{DC2}$ (nM)	Concentration of free drug to increase cDC2 counts to 50% $Vm1_{DC2}$	NA	0.209	Fit from CDX-301 data (5, 6)
del_{DC2} (h^{-1})	Transit rate between cDC2 compartments	NA	0.053	Fit from CDX-301 data (5, 6)
$n1_{DC2}$	Hill coefficient of free drug effect on cDC2 expansion	NA	0.888	Fit from CDX-301 data (5, 6)
$kdeg2_{DC}$ (h^{-1})	Second-order apoptosis rate of cDC2	NA	3.64E-04	Fit from CDX-301 data (5, 6)
f_{DC1}	Fractional rate of cDC1 second-order apoptosis rate compared to that of DC2	NA	0.476	Fit from CDX-301 data (5, 6)

References

1. Satyamitra, M.; Cary, L.; Dunn, D.; Holmes-Hampton, G.P.; Thomas, L.J.; Ghosh, S.P.; CDX-301: a novel medical countermeasure for hematopoietic acute radiation syndrome in mice. *Sci Rep*. **2020**, 10, 1757. https://doi.org/10.1038/s41598-020-58186-1.
2. Cao, Y.; Balthasar, J.P.; Jusko, W.J. Second-generation minimal physiologically-based pharmacokinetic model for monoclonal antibodies. *J. Pharmacokinet. Pharmacodyn.* **2013**, *40*, 597–607. https://doi.org/10.1007/s10928-013-9332-2.
3. Cao, Y.; Jusko, W.J. Incorporating target-mediated drug disposition in a minimal physiologically-based pharmacokinetic model for monoclonal antibodies. *J. Pharmacokinet. Pharmacodyn.* **2014**, *41*, 375–387. https://doi.org/10.1007/s10928-014-9372-2.
4. Verstraete, K.; Vandriessche, G.; Januar, M.; Elegheert, J.; Shkumatov, A.V.; Desfosses, A.; Craenenbroeck, K.V.; Svergun, D.I.; Gutsche, I.; Vergauwen, B.; et al. Structural insights into the extracellular assembly of the hematopoietic Flt3 signaling complex. Blood. **2011**, 118, 60-68. https://doi.org/10.1182/blood-2011-01-329532.
5. Anandasabapathy, N.; Breton, G.; Hurley, A.; Caskey, M.; Trumpfheller, C.; Sarma, P.; Pring, J.; Pack, M.; Buckley, N.; Matei, I.; et al. Efficacy and safety of CDX-301, recombinant human Flt3L, at expanding dendritic cells and

hematopoietic stem cells in healthy human volunteers. *Bone Marrow Transpl.* **2015**, *50*, 924–930. https://doi.org/10.1038/bmt.2015.74.
6. Maraskovsky, E.; Daro, E.; Roux, E.; Teepe, M.; Maliszewski, C.R.; Hoek, J.; Caron, D.; Lebsack, M.E.; McKenna, H.J. In vivo generation of human dendritic cell subsets by Flt3 ligand. *Blood* **2000**, *96*, 878–884.
