# Supplementary material for: A Minimal PBPK/PD Model with Expansion-Enhanced Target-Mediated Drug Disposition to Support a First-in-Human Clinical Study Design for a FLT3L-Fc Molecule
Source: Pharmaceutics. 2024 May 15;16(5):660. doi: 10.3390/pharmaceutics16050660 (PMC11125320; doi:10.3390/pharmaceutics16050660)
Supplement: Supplementary file 1 [file pharmaceutics-16-00660-s001.zip › FLt3L_Fc_manuscript_pharmaceutics_supp-007-gQSPsim - Done.pdf]

Supplemental Instructions of gQSPsim File

Requirements:

Session folder contains the following files:

- SimBiology® project file: Model_FLT3L_FC_manuscript.sbproj
- gQSPSim project file: gQSPSIM_FLT3L_FC_manuscript.sbproj
- Dataset files: DataCynoPK_FLT3L_FC_manuscript.xlsx

Instructions for session file set-up:

- Open the gQSPSim App
- Click on File > Open
- Click on gQSPSim project file: gQSPSIM_FLT3L_FC_manuscript.sbproj
- For Root Directory, click on the Folder symbol and select the folder that contains the model and data files named ‘FLT3L_FC_manuscript’
- ‘Objective Functions Directory’, ‘User-defined Functions Directory’, and ‘Autosave Directory’ default to the same location as that of the root directory
- Select ‘Autosave options’ as suited and click ‘OK’

Instructions for Running Simulation:

- <u>*Running a Simulation*</u>
  - Left click on plus side next to ‘Simulations’ in the left panel
  - Left click on plus side next to ‘Simulations’ for a dropdown list of figure simulations
  - Left click on desired figure simulation (ex: Fig2a_cyno_FLT3LFc_PK)
  - Click the ‘Play’ icon 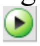 to run the simulation
  - After the simulation runs successfully, click the ‘Graph’ icon 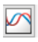 to visualize the simulation
  - To edit other plot properties, click the plot settings button 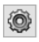

gQSP Simulations:

- “Fig2A_cyno_FLT3LFc_PK” and “Fig2B_cyno_FLT3LFc_PK” will simulate model fits for plasma FLT3L-Fc concentrations in cynomolgus monkey.
- “Fig3_cyno_FLT3LFc_RO” will simulate projected target expression and receptor occupancy for cynomolgus monkey following exposure to FLT3L-Fc.

- “Fig4A-B_human_CDX_PK-DC” and “Fig4C-D_human_CDX_DCtotal” will simulate plasma drug concentrations, DC1, and total DC counts in healthy human volunteers following treatment with FLT3L.
- “Fig5_human_FLT3LFc_PK” will simulate plasma drug concentrations in healthy human volunteers following treatment with FLT3L-Fc using target concentration estimated from cynomologus monkey or healthy volunteers.
- “Fig6A-C_human_FLT3LFc_cynolike” and “Fig6D-F_human_FLT3LFc_humanlike” will simulate projected plasma drug concentrations, activated cDC1, and total DC cells in patients treated with different dosing regimens of FLT3L-Fc using target concentration estimated from cynomologus monkey or healthy volunteers.
- “FigS1A-C_human_FLT3LFc_cynolike” and “FigS1D-F_human_FLT3LFc_humanlike” will simulate projected time profiles of receptor occupancy in patients treated with different dosing regimens of FLT3L-Fc using target concentration estimated from cynomologus monkey or healthy volunteers.
- “FigS2A-C_human_FLT3LFc_cynolike” and “FigS2D-F_human_FLT3LFc_humanlike” will simulate projected time profiles of plasma drug concentrations, activated DC1, and total DC cells in patients treated with different single dose regimens of FLT3L-Fc.
