# Supplementary material for: A Minimal PBPK/PD Model with Expansion-Enhanced Target-Mediated Drug Disposition to Support a First-in-Human Clinical Study Design for a FLT3L-Fc Molecule
Source: Pharmaceutics. 2024 May 15;16(5):660. doi: 10.3390/pharmaceutics16050660 (PMC11125320; doi:10.3390/pharmaceutics16050660)
Supplement: Supplementary file 1 [file pharmaceutics-16-00660-s001.zip › FLt3L_Fc_manuscript_pharmaceutics_supp-008-ODEs.pdf]

Supplemental ODEs and Repeated Assignments

Supplemental ODEs:

d(AmtCentral_ug)/dt = -((kon1_1nMh*ConcCentral_Fc_nM*TargetCentral_nM-kon1_1nMh*KD1_nM*Complex_SB_Central_nM)*Vplasma_L*MWab_ugnmole) - ((1-sig_tight)*L_tight_Lh*ConcCentral_ugL) - ((1-sig_leaky)*L_leaky_Lh*ConcCentral_ugL) + (L_Lh*AmtLymph_ug/Vlymph_L) - (CLp_Lh*ConcCentral_ugL) + ((kabs_CDX_1h*SCdepot_CDX_ug)*min_PBPK) + (infusion_Fc_ug/inf_time_h)

d(AmtTight_ug)/dt = ((1-sig_tight)*L_tight_Lh*ConcCentral_ugL) - ((1-sig_lymph)*L_tight_Lh*ConcTight_ugL) - ((kon1_1nMh*ConcTight_nM*TargetTight_nM-kon1_1nMh*KD1_nM*ComplexTight_nM)*Vtight_L*MWab_ugnmole)

d(AmtLeaky_ug)/dt = ((1-sig_leaky)*L_leaky_Lh*ConcCentral_ugL) - ((1-sig_lymph)*L_leaky_Lh*ConcLeaky_ugL) - ((kon1_1nMh*ConcLeaky_nM*TargetLeaky_nM-kon1_1nMh*KD1_nM*ComplexLeaky_nM)*Vleaky_L*MWab_ugnmole)

d(AmtLymph_ug)/dt = ((1-sig_lymph)*L_tight_Lh*ConcTight_ugL) + ((1-sig_lymph)*L_leaky_Lh*ConcLeaky_ugL) - (L_Lh*AmtLymph_ug/Vlymph_L)

d(TargetCentral_nM)/dt = 1/min_PBPK*(((ksyn_central_nMh)*min_PBPK) - ((kdeg_central_1h*TargetCentral_nM)*min_PBPK) - ((kon1_1nMh*ConcCentral_Fc_nM*TargetCentral_nM-kon1_1nMh*KD1_nM*Complex_SB_Central_nM)*min_PBPK) - ((kon2_1nMh*Complex_SB_Central_nM*TargetCentral_nM-kon2_1nMh*KD2_nM*Complex_DB_Central_nM)*min_PBPK) + (0*Target_Tot_nM*vm_prolif*((RO_SB/100)^alpha)/((RO_SB/100)^alpha+km_prolif^alpha)) + (Target_Tot_nM*vm_prolif*((RO_DB/100)^alpha)/((RO_DB/100)^alpha+km_prolif^alpha)))

d(TargetLeaky_nM)/dt = 1/min_PBPK*(((ksyn_leaky_nMh)*min_PBPK) - ((kdeg_leaky_1h*TargetLeaky_nM)*min_PBPK) - ((kon1_1nMh*ConcLeaky_nM*TargetLeaky_nM-kon1_1nMh*KD1_nM*ComplexLeaky_nM)*min_PBPK))

d(TargetTight_nM)/dt = 1/min_PBPK*(((ksyn_tight_nMh)*min_PBPK) - ((kdeg_tight_1h*TargetTight_nM)*min_PBPK) - ((kon1_1nMh*ConcTight_nM*TargetTight_nM-kon1_1nMh*KD1_nM*ComplexTight_nM)*min_PBPK))

d(Complex_SB_Central_nM)/dt = 1/min_PBPK*(((kon1_1nMh*ConcCentral_Fc_nM*TargetCentral_nM-kon1_1nMh*KD1_nM*Complex_SB_Central_nM)*min_PBPK) - ((kint_1h*Complex_SB_Central_nM)*min_PBPK) - ((kon2_1nMh*Complex_SB_Central_nM*TargetCentral_nM-kon2_1nMh*KD2_nM*Complex_DB_Central_nM)*min_PBPK))

d(ComplexLeaky_nM)/dt = 1/min_PBPK*(((kon1_1nMh*ConcLeaky_nM*TargetLeaky_nM-kon1_1nMh*KD1_nM*ComplexLeaky_nM)*min_PBPK) - ((kint_1h*ComplexLeaky_nM)*min_PBPK))

d(ComplexTight_nM)/dt = 1/min_PBPK*(((kon1_1nMh*ConcTight_nM*TargetTight_nM-kon1_1nMh*KD1_nM*ComplexTight_nM)*min_PBPK) - ((kint_1h*ComplexTight_nM)*min_PBPK))

d(Complex_DB_Central_nM)/dt = 1/min_PBPK*(((kon2_1nMh*Complex_SB_Central_nM*TargetCentral_nM-kon2_1nMh*KD2_nM*Complex_DB_Central_nM)*min_PBPK) - ((kint_1h*Complex_DB_Central_nM)*min_PBPK))

d(AUC_Flt3Fc_nMh)/dt = 1/min_PBPK*((ConcCentral_Fc_nM))

d(AUC_CDX_nMh)/dt = 1/min_PBPK*((CenConc_CDX_nM))

d(PD_DC1)/dt = 1/min_PBPK*((kdeg_DC1_1h*init_DC1) - ((kdeg_DC1_1h*PD_DC1+f_DC1*kdeg2_DC_1h*max(PD_DC1-init_DC1,0)^2/init_DC1)) + ((PD_DC1*kdeg_DC1_1h*vm1_DC1*real(max(0,C3_DC1)^n1_DC1)/(real(max(0,km1_DC1)^n1_DC1)+real(max(0,C3_DC1)^n1_DC1)))))

d(C1_DC1)/dt = 1/min_PBPK*((del_DC1*(C_CDX_FC-C1_DC1)))

d(C2_DC1)/dt = 1/min_PBPK*((del_DC1*(C1_DC1-C2_DC1)))

d(CenAmt_CDX_ugkg)/dt = 1/min_PBPK*(-(CL_CDX_mLhkg*CenConc_CDX_ugmL) - (Vm_CDX_ughkg*CenConc_CDX_ugmL/(Km_CDX_ugmL+CenConc_CDX_ugmL)) + ((kabs_CDX_1h*SCdepot_CDX_ugkg)*min_PBPK))

d(SCdepot_CDX_ugkg)/dt = 1/min_PBPK*(-((kabs_CDX_1h*SCdepot_CDX_ugkg)*min_PBPK))

d(SCdepot_CDX_ug)/dt = 1/min_PBPK*(-((kabs_CDX_1h*SCdepot_CDX_ug)*min_PBPK))

d(C2_DC2)/dt = 1/min_PBPK*((del_DC2*(C1_DC2-C2_DC2)))

d(C1_DC2)/dt = 1/min_PBPK*((del_DC2*(C_CDX_FC-C1_DC2)))

d(PD_DC2)/dt = 1/min_PBPK*(((PD_DC2*kdeg_DC2_1h*vm1_DC2*real(max(0,C3_DC2)^n1_DC2)/(real(max(0,km1_DC2)^n1_DC2)+real(max(0,C3_DC2)^n1_DC2)))) - ((kdeg_DC2_1h*PD_DC2+kdeg2_DC_1h*max(PD_DC2-init_DC2,0)^2/init_DC2)) + (kdeg_DC2_1h*init_DC2))

d(AUC_Flt3Fc_ugmLh)/dt = 1/min_PBPK*((ConcCentral_ugmL))

d(infusion_Fc_ug)/dt = 1/min_PBPK*(-(infusion_Fc_ug/inf_time_h))

d(AUC_CDX_ugmL)/dt = 1/min_PBPK*((CenConc_CDX_ugmL))

d(AUC_RO)/dt = 1/min_PBPK*((RO))

Repeated Assignments:

PD_DC2_foldexpansion = PD_DC2/init_DC2

PD_DC1_foldexpansion = PD_DC1/init_DC1

PD_DCtotal = PD_DC1 + PD_DC2

PD_DCtotal_foldexpansion = PD_DCtotal/(init_DC1+ init_DC2)

PD_DC2_norm = PD_DC2/init_DC2

C3_DC2 = max(C2_DC2,1e-6)

min_PBPK.ConcCentral_ngmL = AmtCentral_ug/Vplasma_L

CenConc_CDX_ugmL = CenAmt_CDX_ugkg/V1_CDX_mLkg

CenConc_CDX_ngmL = CenAmt_CDX_ugkg*1000/V1_CDX_mLkg

PD_DC1_norm = PD_DC1/init_DC1

C3_DC1 = max(C2_DC1,1e-6)

CenConc_CDX_nM = min_PBPK.CenConc_CDX_ngmL*1000/MWCDX_ngnmole

kint_1h = kdeg_central_1h

ConcCentral_ugmL = AmtCentral_ug/Vplasma_L/1000

Target_Tot_nM = TargetCentral_nM+Complex_SB_Central_nM+2*Complex_DB_Central_nM

RO_SB = Complex_SB_Central_nM/max(Target_Tot_nM,1e-18)*100

RO_DB = 2*Complex_DB_Central_nM/max(Target_Tot_nM,1e-18)*100

RO = (1-TargetCentral_nM/max(Target_Tot_nM,1e-18))*100

ConcLeaky_ugL = AmtLeaky_ug/Vleaky_L

ConcLeaky_nM = ConcLeaky_ugL*(1/MWab_ugnmole)

ConcTight_ugL = AmtTight_ug/Vtight_L

ConcTight_nM = ConcTight_ugL*(1/MWab_ugnmole)

ConcCentral_ugL = AmtCentral_ug/Vplasma_L

ConcCentral_Fc_nM = ConcCentral_ugL*(1/MWab_ugnmole)

C_CDX_FC = (FC_flag==0)*CenConc_CDX_nM+(FC_flag==1)*ConcCentral_Fc_nM
